# Non-Conventional Yeasts Whole Cells as Efficient Biocatalysts for the Production of Flavors and Fragrances

**DOI:** 10.3390/molecules200610377

**Published:** 2015-06-04

**Authors:** Luca Forti, Simone Di Mauro, Maria Rita Cramarossa, Sara Filippucci, Benedetta Turchetti, Pietro Buzzini

**Affiliations:** 1 Department of Life Sciences, University of Modena & Reggio Emilia, via G. Campi 103, Modena 41125, Italy; E-Mail: mariarita.cramarossa@unimore.it; 2 Department of Agricultural, Environmental and Food Sciences, Industrial Yeasts Collection DBVPG, University of Perugia, Borgo XX Giugno 74, Perugia 06121, Italy; E-Mails: dimaurosimone@libero.it (S.D.M.); sara.filippucci87@virgilio.it (S.F.); benedetta.turchetti@unipg.it (B.T.)

**Keywords:** non-conventional yeasts, biocatalysis, flavors, fragrances

## Abstract

The rising consumer requests for natural flavors and fragrances have generated great interest in the aroma industry to seek new methods to obtain fragrance and flavor compounds naturally. An alternative and attractive route for these compounds is based on bio-transformations. In this review, the application of biocatalysis by Non Conventional Yeasts (NCYs) whole cells for the production of flavor and fragrances is illustrated by a discussion of the production of different class of compounds, namely Aldehydes, Ketones and related compounds, Alcohols, Lactones, Terpenes and Terpenoids, Alkenes, and Phenols.

## 1. Introduction

Yeasts are key players of a huge variety of traditional and innovative processes (including the production of conventional fermented foods and beverages) and of other high-value bulk and fine chemicals. Although a number of sutdies are still waiting to be developed into profitable ventures, because they are still confined to the laboratory scale, many other products obtained by yeast metabolism are commercially produced. In this framework, the impact of yeast biotechnology on human activities has been extensively documented [1,2,3,4]. Much of this research is heavily related to the model yeast *Saccharomyces cerevisiae* (otherwise labeled as baker’s yeast). This yeast species is traditionally used for producing bread, beer, wine, and some ethnic fermented foods and beverages in Asia, Africa and South America [2]. More recently, *S. cerevisiae* has also been used in the biofuel industry (e.g., ethanol) and for the production of heterologous compounds, such as recombinant proteins, human insulin, hepatitis and human papillomavirus vaccines [4,5,6].

This plethora of studies and industrial applications has determined that to most people (including a number of microbiologists) yeasts are exemplified by the species *S. cerevisiae*. This is in spite of the fact that this domesticated microorganism represents only a modest fragment of the vast biodiversity and biotechnological potential occurring in the yeast world. Actually, in recent decades the taxonomic and metabolic diversity of the so-called “Non-Conventional Yeasts” (NCYs) have revealed innumerable promising biotechnological properties [7,8,9]. There is no generally accepted definition of NCYs. Even though a number of scientists include a few additional species (namely, at least *Schizosaccharomyces pombe* and *Kluyveromyces lactis*) into the group of “conventional yeasts” (CYs), many other consider NCYs as synonymous to “non-*Saccharomyces*” yeasts. Overall, Sibirny and Scheffers [8] underlined that, since an increasing number of NCYs is gaining importance in fundamental and applied microbiological sciences, the term NCYs is gradually losing significance and usefulness. Beyond any definition, NCYs represent the vast majority of yeast species so far described: really, the current description of yeast taxonomy accounts for more than 130 ascomycetous and basidiomycetous genera and over 1600 species [10]. Undoubtedly, this enormous yeast diversity includes many species possessing useful, and sometimes uncommon, metabolic features potentially interesting for both food and non-food industries [4,8,11].

Despite the increasing use of NCYs in biotechnology, there are still significant opportunities for improving their exploitation. Demands for increased productivity from wider substrate range, production of novel compounds at the industrial level, as well as changing of consumer preferences, can lead to a great interest in further enhancing the number of NCYs being used by selecting (or developing) strains with novel and attractive properties [6].

Flavors play a very important role in the quality perception of food and beverages, whereas fragrances represent an important part of the soap and perfume industry. For many years, the extraction from natural sources or the use of traditional fermentation processes were the sole methods for obtaining this type of compounds. Chemical synthesis still represents the cheaper technology for their production. However, the rising consumer requests for natural flavors and fragrances have encouraged a growing part of the scientific community to develop novel biocatalysts for producing these molecules [12,13,14]. Although the biocatalytic ability of yeasts is a well-known phenomenon, and a few processes used yeast whole cells for selected biotransformation at the industrial level [4], the studies aimed at evaluating the potential of NCYs as biocatalysts has never been substantially reviewed so far.

In this review, we will consider the application of NCYs for producing flavors and fragrances via biocatalysis. Due to the fast increase in the number of described yeast species and to the formal reassignment to some old synonyms to new taxa, many species names cited in current literature have become outdated. Accordingly, all original taxonomic designations reported in the cited references were checked and, if necessary, updated according to the latest yeast taxonomic guidelines reported in Kurtzman *et al.* [10].

## 2. Biocatalytic Production of Flavors and Fragrances by NCYs

### 2.1. Definition of Biocatalysis and Their Impact on the Production of Flavors and Fragrances

Biocatalysis sums up both biotrasformations (*i.e.*, the conversion of defined substrates using whole cell or resting cell systems) and enzyme catalysis (*i.e.*, the conversion of defined substrates using cell-free extracts or purified enzymes) and may be defined as a process that describes a reaction, or a set of simultaneous reactions, in which a pre-formed precursor molecule is converted using enzymes and/or whole cells, or combinations thereof, either free or immobilized [15].

Biocatalytic processes are increasingly used for replacing conventional chemical processes and to make possible the formation of new products. Among the reasons justifying the use of biocatalysts, the high specificity and selectivity, and the possibility to use “environmental-friendly” conditions (e.g., solvent-free approaches, low working temperatures and pressures, and waste reduction) represent, undoubtedly, the most attractive ones. Furthermore, biocatalysis can sometimes allow reduction of the number of steps in a synthetic route [16,17,18,19].

Although both isolated whole cells and enzymes can be used as biocatalysts, whole cells are often preferable because they are more convenient and stable sources than purified enzymes, with no need for costly purification and coenzyme addition. In fact, although enzymes are considered as powerful tools, the number of enzymes commercially available on the market (free or immobilized) is still quite limited, particularly for some types of uncommon substrates, namely some compounds of pharmaceutical interest. Moreover, when the enzymes are kept within their natural environment (whole cell cytoplasm) lesser inactivation usually occurs [20]. On the contrary, in case of single step biotransformation, isolated enzymes can actually be considered the best choice, and the application of immobilized enzymes, either in aqueous or in organic media, could increase the biocatalytic productivity.

At present, the increasing use of biotransformations for producing flavors and fragrances is essentially justified by two reasons. Firstly, biocatalysis sometimes allow the production of regio- and stereoselective compounds under mild conditions. Secondly, the use of cell biocatalysts is unanimously considered a lesser pollutant (solvent-free) technology. Accordingly, molecules obtained by such bioprocesses can be labeled as natural; in fact, both US and EU laws have already labeled them as natural flavor, and all volatile organic compounds (VOCs) obtained from living cells, including microorganisms, are labeled as GRAS (Generally Recognized As Safe) [13]. As a result, the biocatalytic potential of food-grade microorganisms (in particular, the use of whole cells for obtaining specific molecules) has attracted considerable interest.

### 2.2. Aldehydes, Ketones and Related Compounds

Within the last five years, a significant and growing number of ketoreductases isolated from NCYs have become available [19,21]. The broad substrate range and high selectivity exhibited by these enzymes repeatedly outperform other ketone reduction chemistries, making biocatalysis the general method of choice for ketone reductions [21]. A screening identified the species *Candida sorbophila*, which, after extensive optimization, produced the (*R*)-hydroxy amide in high enantiomeric excess (>98%) [22].

A more recent study reported the expression of a fatty acid hydroperoxide lyase (HPO lyase) from green bell pepper in *Yarrowia lipolytica* cells [23]. A POX2 (promoter of the *Y. lipolytica* acyl-CoA oxidase 2 gene inducible by fatty acid) expression system was constructed for multiple copy integration into the *Y. lipolytica* genome. This allowed the introduction of multiple HPO lyase genes into the genome of this species for increasing its aptitude towards fed-batch cultivation and inducing highest production of HPO lyase activity. These studies demonstrated the ability of *Y. lipolytica* to be used as a useful host for producing high quantities of C6-aldehydes via biocatalysis [23,24].

### 2.3. Alcohols

The natural aroma chemicals 2-phenylethanol (2-PE) and 2-phenylethylacetate (2-PEAc) are widely applied in the cosmetic, perfume, and food industries and are mainly produced by chemical synthesis [25]. Alternatively, they can be produced from L-phenylalanine via biocatalysis catalyzed by NCYs whole cells [26,27,28]. Batch and fed-batch processes were approached using *Kluyveromyces marxianus* whole cells: 5.23 and 26.5 g/L of 2-PE, and 5.85 and 6.1 g/L of 2-PEAc were respectively obtained. The use of an organic phase (with no complex *in situ* product removal and feeding techniques or costly process modifications) made fed-batch approach an interesting alternative to current industrial processes [26,27]. More recently, Gao and Daugulis [28] used a solid-liquid two-phase partition bioreactor system (where polymer beads act as sequestering immiscible phase, thus reducing the aqueous 2-PE concentration to non-inhibitory levels) as an *in situ* product removal technique. A final 2-PE concentration of 20.4 g/L was achieved (with 1.4 g/L in the aqueous and 97 g/L in the polymer phase) [28]. An alternative method for producing 2-PE from L-phenylalanine via biocatalysis was the Ehrlich pathway and considerable progress has been made in the development of this process [25].

In the last fifteen years, a number of papers tried to optimize the conditions of biocatalysis to improve the production of 2-PE with very diverse results (Table 1) [29,30,31,32,33].

**Table 1:** Optimization of 2-phenylethanol (2-PE) production via biotransformations catalyzed by NCYs whole cells.

Species	2-Phenylethanol (2-PE) Production	References
*Kluyveromyces marxianus*	no more than 1.0 g/L	[29]
*Kluyveromyces marxianus*	5.6 g/L (187% than non-optimized medium)	[30]
*Kluyveromyces marxianus*	0.8 g/L (in bioreactor)	[31]
*Pichia fermentans*	0.5 g/L, molar yield 71%	[32]
*Pichia fermentans*	0.5 g/L, molar yield 74%	[33]

The production of other alcohols via biocatalysis was studied. Andreu and Del Olmo [34] described the use of whole cells of *K. marxianus* and *Lindnera jadinii* (formerly *Pichia jardinii*) as biocatalysts for producing the chiral products derived from the aldol reaction between acetone and some aromatic aldehydes, as well as the chiral 1,3-diols deriving from their reduction. The resolution of the racemic starting material and the recovery of the aldol with the *S* configuration was sometime possible using *K. marxianus*. Both species showed complementary enantioselectivity by generating the new stereogenic center through the reduction of the carbonyl moiety, which provides access to three of the four possible diastereomeric diols with high enantiomerical purity. More recently, the same authors used whole cells of *Schwanniomyces etchellsii* (formerly *Debaryomyces etchellsii*) for the production of up to 10 g/L of (*R*)-(−)-phenylacetylcarbinol [(*R*)-PAC] via acyloin condensation with a trace of undesired side products [34]. Likewise, *K. marxianus* was used as biocatalyst to achieve the kinetic resolution of racemic (±)-phenylacetylcarbinol: (*S*)-(+)-phenylacetylcarbinol was obtained with excellent stereo-selectivity. The ketone reduction by *S. etchellsii* of both isolated stereoisomers (*R* and *S*) gave two of the four diastereoisomers of 1-phenyl-1,2-propanediol, which are key precursors of interesting pharmaceutical and chemical products [34]. In this frame, the pH of the reaction medium greatly affected the selectivity of biotransformation of benzaldehyde into (*R*)-PAC or benzyl alcohol: better selectivity (*R*) was achieved under more acidic conditions [34,35,36].

In recent years, a few authors reported the ability of whole cells of *Ogataea glucozyma* (syn. *Pichia glucozyma*) to catalyze the stereoselective reduction of different aromatic ketones and ketoesters to the corresponding (*S*)-alcohols with high yields and enantioselectivity [37]. The biotransformation of ketoesters often occurs with competition between carbonyl reduction and ester hydrolysis. However, this competitive enzymatic activity can be modulated by using appropriate reaction condition and allowed for the preparation of hydroxyesters, hydroxyacids and lactones often in a very selective manner.

### 2.4. Lactones

γ-Lactones are industrially important flavor compounds that are widely distributed in foods, fruits, and beverages and are used in many fruity aromatic foods and cosmetics [38]). Although many synthetic γ-lactones are currently utilized as artificial flavors, the consumer demand towards natural flavors increased the studies aimed at producing γ-lactones via biocatalysis. Natural lactones, such as γ-decalactone and γ-dodecalactone, have been produced from free fatty acids, hydroxyl fatty acids, or oils through several enzymatic steps in the γ-oxidation system of yeasts [39]. Due to the importance of these compounds, some biotransformation process for producing γ-lactones catalyzed either by both CYs and NCYs exhibiting high conversion yields were developed in the last fifteen years [40,41,42].

Among the different lactones, γ-dodecalactone is currently used as an aroma or taste component of consumable materials such as foodstuffs, chewing gums, toothpastes, cosmetic powders, hair preparations, medicinal products, smoking tobaccos, detergents, perfume compositions, and perfumed articles [43,44,45,46,47,48]. A new biotransformation process for the production of γ-dodecalactone from oleic acid was developed by using permeabilized cells of *Lipomyces lipofer* (syn. *Waltomyces lipofer*). A conversion yield of 76% and a productivity of 1.5 g/L·h were obtained [39]. γ-Decalactone production via ricinoleic acid biotransformation was also studied. The species *Sporidiobolus salmonicolor* (formerly *Sporobolomyces odorus*) has been widely reported for its ability to convert castor oil (or its derivatives) to γ-decalactone [49]. The same species was used by Garbe and Tressl [50] to investigate the catabolic pathways (via Baeyer-Villiger-type oxidation) of both γ- and δ-lactones by incubation of whole cells in ^13^C labeled ethyl (±)-5-hydroxydecanoate.

*Y. lipolytica* was widely studied for its capability to produce lactones via biocatalysis. Groguenin *et al.* [51] reported that this species possesses five acyl-CoA oxidases (Aox1p to 5), which catalyze the first reaction of β-oxidation. Accordingly, they constructed a strain lacking this activity, which produced 10 times more γ-decalactone than the wild type. On the contrary, Escamilla-Garcia *et al.* [52] used a Doehlert experimental design to optimize lactone synthesis by *Y. lipolytica*. The accumulation of γ-decalactone was higher (496 mg/L) at pH around 5 and increased at low aeration, while the 3-hydroxy-γ-decalactone accumulation was higher (660 mg/L) at low pH and high aeration conditions. They postulated that β-oxidation fluxes (*i.e.*, the accumulation of 3-hydroxy-γ-decalactone) were strongly oxygen-dependent [53]. More recently, Braga and Belo [54] used *Y. lipolytica* whole cells for the batch and fed-batch production of γ-decalactone from ricinoleic acid. The best γ-decalactone productivity (215 mg/L·h) was obtained with 60 g/L of cells and castor oil concentration.

Other studies carried out in the last fifteen years on the use of NCYs for producing lactones via biocatalysis are reported in Table 2 [55,56,57,58].

**Table 2:** Studies carried out in the last fifteen years on the use of NCYs for producing lactones via biocatalysis.

Species	Substrate	Product	References
*Candida albicans*	Linoleic acid	γ-nonalactone	[55]
*Candida tropicalis*	Linoleic acid	γ-nonalactone	[55]
*Cryptococcus laurentii*	β-carotene	β-ionone, DHA	[56]
*Lindnera saturnus* (formerly *Hansenula saturnus*)	Ricinoleic acid	3-hydroxylactone, decen-4-olides	[57]
*Phaffia rhodozyma*	β-carotene	β-ionone, DHA	[56]
*Yarrowia lipolytica*	Ricinoleic acid	3-hydroxylactone, decen-4-olides	[57]
*Yarrowia lipolytica*	Ricinoleic acid	γ-decalactone	[58]
*Yarrowia lipolytica* (formerly *Candida lipolytica*)	Linoleic acid	γ-nonalactone	[55]

### 2.5. Terpenes and Terpenoids

Terpenes occur widely in nature and are obtained in large scale as industrial residues. Monoterpenes include a number of flavoring compounds (over 400 different naturally occurring structures), and represent a valuable resource for the flavor and fragrance industry. Actually, monoterpene hydrocarbons dominate in the composition of most essential oils. They are usually separated from oils by rectification because of low odor activity, high hydrophobicity and their high tendency to auto-oxidize and polymerize. This renders a few abundant monoterpenes, namely α-pinene and limonene, industrial wastes and inexpensive starting materials for chemical and biochemical transformations. Moreover, the rising consumer requests for natural flavors and fragrances have encouraged the scientific community to develop novel prokaryotic and eukaryotic biocatalysts for producing this class of molecules [59,60]. However, the number of studies involving the biocatalytic ability of both CYs and NCYs for the biotransformation of terpenes represents only a little percentage of the current literature [59].

**Figure 1:**
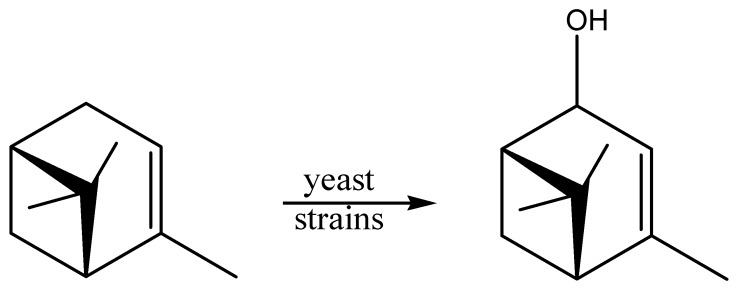
Biotransformation of (−)-α-pinene to verbenol.

Monoterpene precursors can be converted via biotransformation into their more valuable oxygenated derivatives [61]. α-Pinene is a component of the wood and leaf oils of a wide variety of plants and is the principal constituent of turpentine from most conifers [62]. This low-price bicycle monoterpene is largely employed in fragrance and flavor industries as raw material for the synthesis of high-value products [63,64], and is commonly used as substrates for biotransformation. Rottava *et al.* [65] showed that 13 different yeast strains, isolated from orange juice industry residues, soils of citric fruits, and leaves of citric fruits, were able to convert the substrate (−)-α-pinene to verbenol (Figure 1).

Likewise, Javidnia *et al.* [66] studied the biotransformation of α- and β-pinene by seven different microorganisms, including the yeasts *Candida albicans*. The results showed that this species was unable to biotransform α-pinene, and only traces of sabinol, myrtenol and myrtenal were observed in the biotransformation of β-pinene.

**Figure 2:**
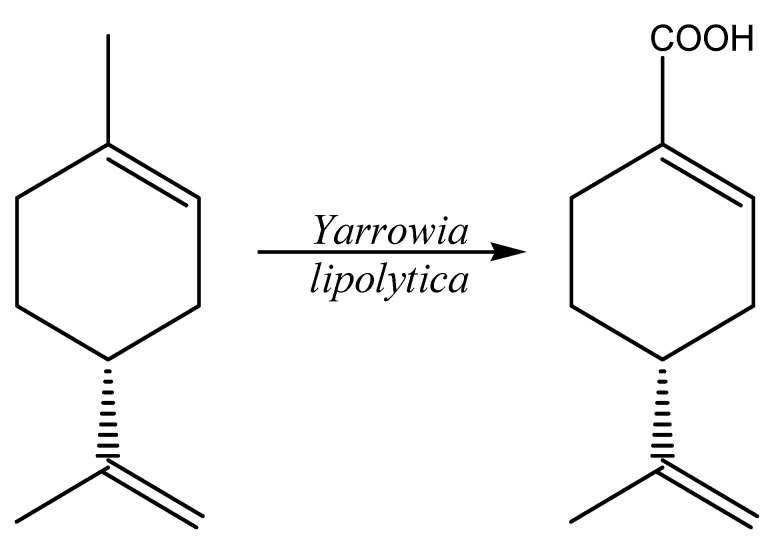
Biotransformation of (+)-limonene to perillic acid promoted by *Y. lipolytica*.

Different NCYs were screened for the biotransformation of (+)-limonene [61]. *Arxula adeninivorans* and *Y. lipolytica* hydroxylated the substrate on the *exo*-cyclic methyl group to give one major products, thus converting (+)-limonene to perillic acid (0.06 and 1.0 g/L; yield 3% and 50%) (Figure 2).

**Figure 3:**
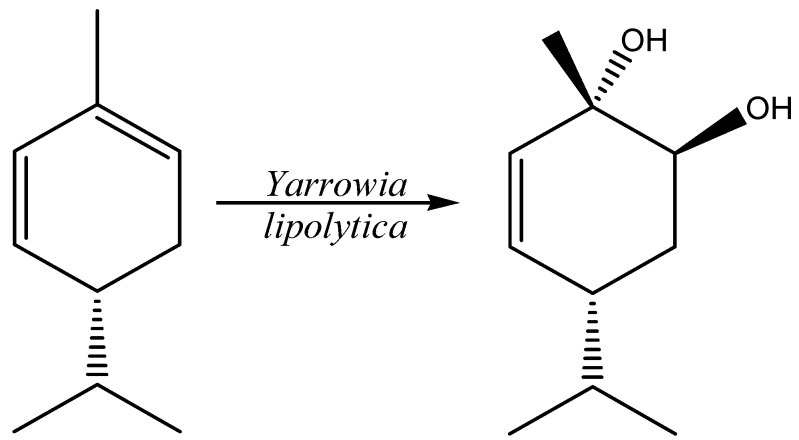
Biotransformation of (−)-(*R*)-α-phellandrene to 5-*p*-Menthene-1,2-diol promoted by *Y. lipolytica*.

More recently, Iscan *et al.* [67] reported the biotransformation of the enantiomerically pure (−)-(*R*)-α-phellandrene with 16 different microorganisms, including some NCYs. 5-*p*-Menthene-1,2-diol was identified as the major product (yields 16%) for the biotransformation promoted by *Y. lipolytica* (Figure 3).

**Figure 4:**
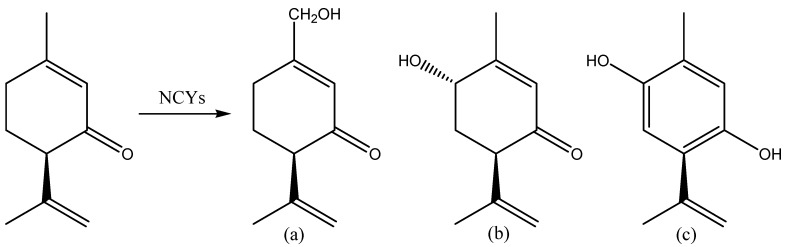
NCYs catalyzed biotransformation of (−)-piperitone to (4*R*)-7-hydroxy-piperitone (**a**); (4*R*,6*S*)-*trans*-6-hydroxy-piperitone (**b**) and 2-isopropyl-5-methyl-hydroquinone (**c**).

The microbial and enzymatic biotransformation of some monoterpenoids into highly valuable flavoring derivatives is also has increasing interest because of their economic potential for the perfume, soap, food, and beverage industry [14,68]. In particular, the bioconversion of carvone, myrtenal and geraniol has been studied. Biocatalytic transformation of carvones and other terpene ketones has been the focus of some studies [69,70,71], reporting that different NCYs may catalyze the reduction of C=C and C=O double bonds competitively, affording a mixture of saturated ketones, saturated alcohol and, more rarely, the allylic alcohol*.* Ascomycetous and basidiomycetous NCYs, belonging to species of the genera *Dekkera*, *Eremothecium*, *Geotrichum*, *Hanseniaspora*, *Kloeckera*, *Kluy*v*eromyces*, *Lipomyces*, *Metschnikowia*, *Pichia*, *Rhodotorula*, *Schwanniomyces*, *Sporodiobolus*, *Torulaspora*, *Trichosporon*, and *Yarrowia*, were screened since the 1990s for the their ability to catalyze the biotransformation of monoterpenoid ketones [69]. A relatively small number of NCYs gave hydroxylation of (−)-piperitone to different products (7-hydroxy-piperitone, trans-6-hydroxy-piperitone and 2-isopropyl-5-methyl-hydroquinone, Figure 4), with yields ranging between 8% and 60% (corresponding to product concentrations of 0.04 to 0.3 g/L, respectively). None of the NCYs tested reduced (−)-piperitone. These results are in accordance with those reported by van Rensburg *et al.* [61].

**Figure 5:**
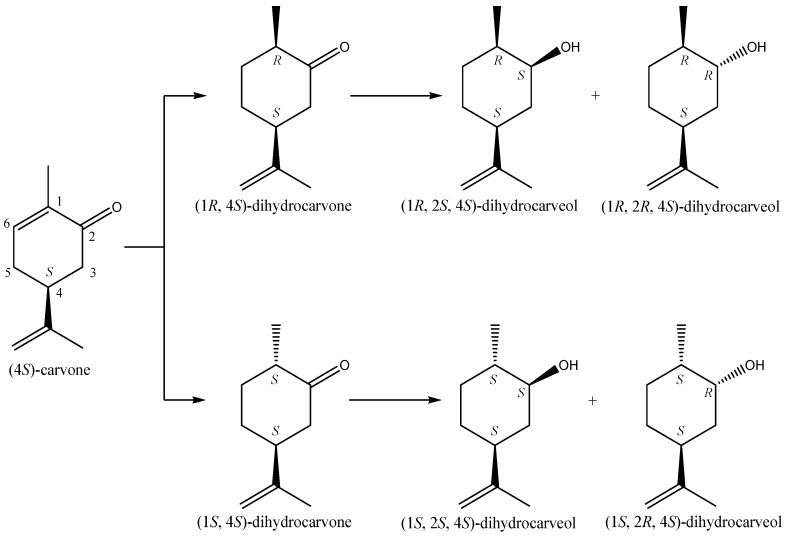
Bioconversion pathway of (4*S*)-carvone by NCYs.

**Figure 6:**
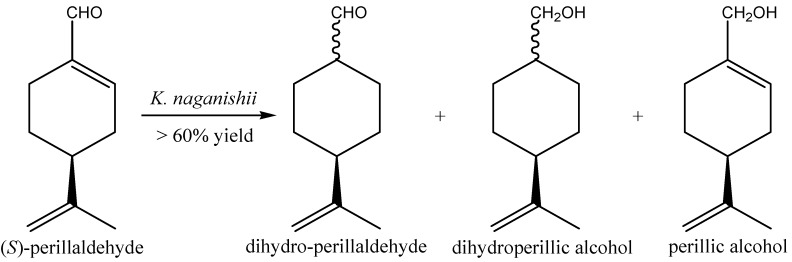
Bioconversion of (*S*)-perillaldehyde by NCY *K. naganishii*.

In contrast, almost all NCYs tested gave reduction of carvone, but with different enzyme activity (Figure 5 and Figure 6). Reduction of (4*R*)-carvone was often much faster than reduction of (4*S*)-carvone, and yields of up to 90% were obtained within 2 h. Some NCYs only reduced the C=C to yield the dihydrocarvone isomers with the stereochemistry at C-1 always *R*, while others also reduced the ketone to give the dihydrocarveols with the stereochemistry at C-2 always *S* for (4*R*)-carvone, but sometimes *S* and sometimes *R* for (4*S*)-carvone.

Carballeira *et al.* [72] found that the reduction of (4*R*)- or (4*S*)-carvones catalyzed by *Schizosaccharomyces octosporus* whole cells showed that the stereochemistry of the ketones determined the reduction pathway (Figure 5). Indeed, while (4*S*)-carvone yields (2*R*,4*S*)-carveol (50% yield), the (4*R*)-carvone carries to (1*R*,2*S*,4*R*)-dihydrocarveol (80% yield) as the major product. In the same way, Goretti *et al.* [70] used a set of environmental NCYs lyophilized whole cells as biocatalysts for the reduction of (4*S*)-carvonein in the presence of glucose (acting as auxiliary substrate for cofactor-recycling system). Interestingly, the species *Lindnera amylophila* and *Kazachstania naganishii* (formerly *Pichia amylophila* and *Saccharomyces naganishii*, respectively) exhibited a bioconversion near to 100%. However, their biocatalytic activity was quite different. In the first case, a mixture of (1*R*,2*S*,4*S*)- and (1*S*,2*S*,4*S*)-dihydrocarveol are produced as the major compounds (25.3% and 63%, respectively), by the consecutive reduction of C-C and C-O double bonds, respectively; on the contrary, the cells of *L. naganishii* catalyzed almost exclusively the reduction of the carbon-carbon double bond of (4*S*)-carvone affording (1*S*,4*S*)- and (1*R*,4*S*)-dihydrocarvones (44.4% and 39.7%, respectively) as major products (Figure 5).

An optimization strategy for asymmetric bioreduction of (4*S*)-carvone by *Cryptococcus gastricus* was recently reported [71]. Response surface methodology was used for simultaneously maximize the bioreduction yield of (4*S*)-(+)-carvone (>95%) by whole cells of *C. gastricus* and minimize the incidence of side reactions (<1%). More recently, the bioreduction of (4*R*)-carvone by NCYs whole cells of the genera *Candida*, *Cryptococcus*, *Debaryomyces*, *Hanseniaspora*, *Kazachstania*, *Kluyveromyces*, *Lindnera*, *Nakaseomyces*, *Vanderwaltozyma* and *Wickerhamomyces* has been studied [73]. The prevalent catalytic activity was the ene-reductase(ER)-catalyzed reduction of the substrate into a mixture of (1*R*,4*R*)- and (1*S*,4*R*)-dihydrocarvone, with a clear-cut preference towards the production of (1*R*,4*R*)-diastereomer. Only traces of dihydrocarveols, derived from the subsequent carbonyl reductase-catalyzed reduction of the carbonyl group were found. *Hanseniaspora guilliermondii* exhibited a good bioconversion yield (about 63%), coupled with an excellent selectivity (diastereomeric excess (d.e.) = 98%), compatible with its application as potential source of dihydrocarvone.

The biotransformation of some terpene aldehydes ((*S*)-perillaldehyde and the aromatic terpene aldehyde α-methyl-cinnamaldehyde) by a set of NCYs belonging to 21 species of the genera *Candida*, *Cryptococcus*, *Debaryomyces*, *Hanseniaspora*, *Kazachstania*, *Kluyveromyces*, *Lindnera*, *Nakaseomyces*, *Vanderwaltozyma*, and *Wickerhamomyces*has has recently been reported [74]. NCYs whole cells exhibited a moderate ability to reduce the monocyclic (*S*)-perillaldehyde: *K. naganishii* exhibited bioconversion yields higher than 60% (Figure 6). (*S*)-Perillaldehyde was initially reduced to dihydro-perillaldehyde and successively to dihydroperillic alcohol. Interestingly, the same species catalyzed the direct reduction of the carbonyl group of (*S*)-perillaldehyde, which led to the formation of perillic alcohol.

**Figure 7:**
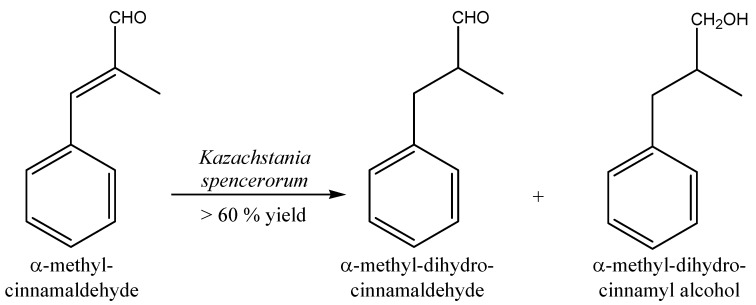
Bioconversion of α-methyl-cinnamaldehyde by NCY *Kazachstania spencerorum*.

On the contrary, NCYs whole cells poorly reduced α-methyl-cinnamaldehyde: only *Kazachstania spencerorum* exhibited bioconversion yields higher than 60%. α-Methyl-cinnamaldehyde was initially reduced to α-methyl-dihydro-cinnamaldehyde, while the subsequent reduction of the carbonyl group led to the formation of α-methyldihydrocinnamyl alcohol (Figure 7).

**Figure 8:**
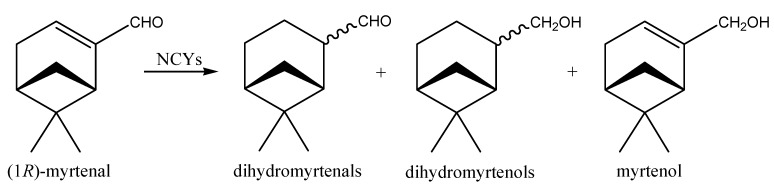
Bioconversion of (1*R*)-myrtenal by whole-cells of NCYs.

The biotransformation of the α,β-unsaturated aldehyde (1*R*)-myrtenal catalyzed by NCYs whole cells was also investigated [73]. Overall, NCYs showed good (occasionally excellent) aptitudes to biotransform (1*R*)-myrtenal into derivative compounds (Figure 8): about one third of strains gave percentage of conversion ≥95%. Among them, *Candida freyschussii* and *K. spencerorum* converted 100% of the precursor. Interestingly, in almost all cases, biocatalytic ability was prevalently related to the activity of carbonyl reductases (CRs) associated to whole-cells, affording myrtenol as the main product of the bioconversions. On the contrary, the results obtained apparently suggest that (1*R*)-myrtenal is not a good substrate for the ER activity. In fact, only a few strains showed prevalent ER-catalyzed asymmetric C=C bioreduction of the α,β-unsaturated aldehyde: they produced dihydromyrtenals, which were then further reduced by CRs to give dihydromyrtenols, that resulted the main products when whole cells of *Cryptococcus albidus* and *L. amylophila* were used as biocatalysts. Although only one study describing the biotransformation of myrtenal by algae has so far been published [75], the industrial importance of its structurally-related monoterpene alcohol derivative myrtenol is becoming greater and greater: this compound is a fragrance ingredient used in decorative cosmetics, fine fragrances, shampoos, toilet soaps and other toiletries as well as in non-cosmetic products such as household cleaners and detergents [76].

**Figure 9:**
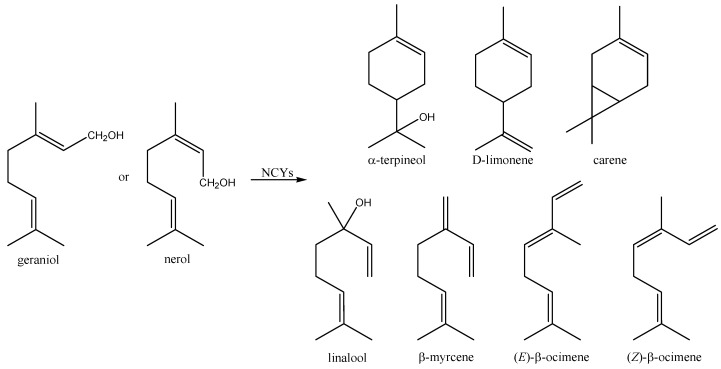
Bioconversion of geraniol and nerol by whole-cells of *Debaryomyces*, *Kluyveromyces*, and *Pichia* NCYs.

Little attention has so far been devoted to the ability of yeasts to convert acyclic monoterpenes (e.g., geraniol and nerol). King and Dickinson [77] observed that both CYs and NCYs produced linalool and α-terpineol from both geraniol and nerol. *Torulaspora delbrueckii* also exhibited the ability to form geraniol from nerol [77]. More recently, Ponzoni *et al.* [78] have screened sixty environmental NCYs belonging to the genera *Debaryomyces*, *Kluyveromyces*, and *Pichia* for their ability to biotransform the acyclic monoterpenes geraniol and nerol (Figure 9). The aptitude to convert both compounds (from 2.6% to 30.6%, and from 2.7% to 29.1% cell dry weight, respectively) was apparently common in NCYs. Depending upon the substrate used, the production of linalool, α-terpineol, β-myrcene, d-limonene, (*E*)-β-ocimene, (*Z*)-β-ocimene, or carene was observed. Linalool was the main product obtained from geraniol, whereas linalool and α-terpineol were the main products obtained through the conversion of nerol. Yet, differently from nerol, the aptitude to exhibit high bioconversion yields of geraniol to linalool was an apparently genus-related character, whereas the ability to produce other monoterpenes was a both genus- and habitat-related character.

### 2.6. Alkenes

The reduction of alkenes is a powerful tool in modern asymmetric synthesis and different approaches are available at the industrial scale [79]. Biotransformation of suitable precursors is an attractive, sustainable and cost-effective alternative to “chemical” reduction: the biocatalytic analog relies on ERs to perform the reduction of activated C=C bonds. ERs are Flavine MonoNucleotide (FMN)-dependent enzymes belonging to the so-called “old yellow enzyme” (OYE) family, and have been intensely investigated over the past years in view of their applicability in preparative-scale biotransformations [80,81,82].

Asymmetric bioreduction of electron-poor alkenes catalyzed by ERs is undoubtedly one of the emerging biocatalysis and represents a key step in the synthesis of increasingly complex drugs and fragrances. The OYE family has rapidly grown and some ER homologues were isolated from different biological sources, namely higher plants [83], bacteria [84,85,86], yeasts, including NCYs [87,88,89], and filamentous fungi [90]. Among CYs, with a few exceptions [87,89,91,92], studies on ER homologues have been performed so far almost exclusively on ERs isolated from *Saccharomyces* spp. yeasts, and in particular from *S. cerevisiae* [93,94,95]. However, in recent years, screenings carried out on a large set of NCYs aimed at selecting whole-cell biocatalysts for the bioreduction of carbonyl activated C=C of (4*S*)-(+)- and (4*R*)-(−)-carvone, and of some terpene aldehydes have been performed, as reported above (terpene Section of this review).

Bioreduction of other carbonyl-activated alkenes to flavoring compounds has also been reported. The bioreduction of the α,β-unsaturated ketones ketoisophorone, 2-methyl- and 3-methyl-cyclopentenone by NCYs belonging to the genera *Candida*, *Cryptococcus*, *Debaryomyces*, *Hanseniaspora*, *Kazachstania*, *Kluyveromyces*, *Lindnera*, *Nakaseomyces*, *Vanderwaltozyma*, and *Wickerhamomyces* has been reported by Goretti *et al.* [74]. Frequently, NCYs whole cells exhibited extremely high (>90% or even 100%) ketoisophorone and 2-methyl-cyclopentenone bioconversion yields after 120 h, via asymmetric reduction of the conjugated C=C bond catalyzed by ERs.

**Figure 10:**
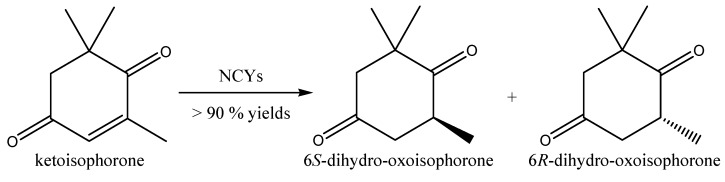
Bioconversion of ketoisophorone by whole-cells of NCYs.

High chemoselectivity due to low competing CRs was also sometimes observed, together with a moderate stereoselectivity for *R*-isomers (% ee for 6*R*-dihydro-oxoisophorone, 6*R*-DOIP, from 10 to 44, Figure 10). After 24 h, *K. spencerorum* converted more than 80% of KIP in 6*R*-DOIP, which represented, at this stage, the almost exclusive product of bioreduction. Interestingly, 6*R*-DOIP is a key aroma constituent of tobacco and saffron [96].

NCYs whole cells showed a moderate ability to bioreduce 2-methyl-cyclopentenone (2-MCPO); *Debaryomyces coudertii*, *Debaryomyces nepalensis* and *K. spencerorum* exhibited bioconversion yields higher than 90%.

**Figure 11:**
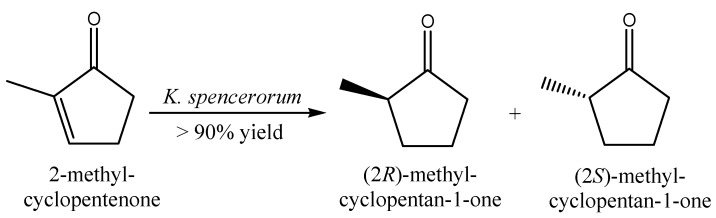
Bioconversion of 2-methyl-cyclopentenone by whole-cells of *K. spencerorum*.

2-MCPO was initially reduced by NCYs whole cells to a mixture of (2*R*)-methyl-cyclopentan-1-one (2*R*-MCPO) and (2*S*)-methyl-cyclopentan-1-one (2*S*-MCPO). The subsequent reduction of the carbonyl group of both 2*R*- and 2*S*-MCPO led to the formation of the corresponding 1*R*,2*R*-, 1*S*-2*R*- 1*R*,2*S*-, and 1*S*,2*S*-methyl-cyclopentan-1-ols. All four of the above-mentioned strains also exhibited a predominant ERs *vs.* CRs activity. In particular, 2*R*- and 2*S*-MCPO were the exclusive products obtained by 2-MCPO reduction catalyzed by *K. spencerorum* (Figure 11). On the whole, NCYs whole cells exhibited a modest preference for the 2*R*-MCPO enantiomer. 2-Methylciclopentanone was described as having a roast beef odor [97]. None of the tested NCYs exhibited the ability to reduce the 3-MCPO, probably because of the presence of a substituent group in the β position of 3-MCPO.

### 2.7. Phenols

It has been described that yeasts can utilize monomeric phenolic compounds [98]. A typical example here is the biosynthesis of vanillin, usually produced by chemical synthesis from guaiacol or via extraction from vanilla beans (natural vanillin content 2%). The trends toward natural flavors and the high price of natural vanillin has driven the search of microbial processes for natural vanillin production. This requires a natural precursor such as ferulic acid, which is present in plant cell walls (e.g., sugar beet pulp, *etc.*).

Though the biotransformation of ferulic acid has been studied extensively to develop a process for the production of vanillin [99], there are other value-added minor metabolites produced during this biodegradation, including 4-vinyl guaiacol, vanillic acid, acetovanillone, vanillyl alcohol, dihydroferulic acid, coniferyl alcohol, dihydroconiferyl alcohol or homovanillic acid [100].

Whole cells of *Debaryomyces hansenii* metabolized ferulic acid to 4-vinyl guaiacol by the non-oxidative decarboxylation of its side chain [101] This biotransformation is a highly value added process as 4-vinyl guaiacol is nearly 40 times costlier than ferulic acid. *D. hansenii* produced 1470 mg/L of vinyl guaiacol in 10 h, corresponding to a molar yield of 95%. However, the production of vanillin from 4-vinyl guaiacol through this biotechnological route is not very economical as the vanillin levels were 169 mg/L at the fifth hour.

**Figure 12:**
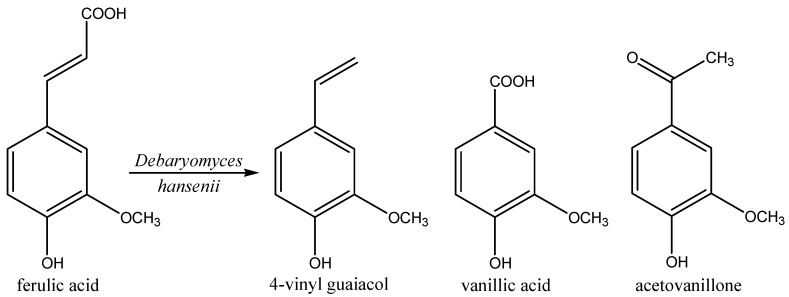
Biotransformation of ferulic acid into higher value added products by *Debaryomyces hansenii*.

Recently, Max *et al.* [102] used a factorial design to optimize the biotransformation of ferulic acid into higher value added products such as 4-vinyl guaiacol, vanillic acid and acetovanillone by *D. hansenii* (Figure 12). Depending on the glucose and nitrogen concentrations, the major degradation products were 1.2 g/L of 4-vinyl guaiacol after 72 h (molar yield of 86.0%), 1.1 g/L of vanillic acid after 360 h (molar yield of 91.1%) or 1.7 g/L of acetovanillone after 408 h (molar yield of 98.8%) in reactions carried out with 2 g/L of ferulic acid. Vanillin, vanillyl alcohol or 4-ethylguaiacol were present in lower amounts. Previous studies have also reported that *Rhodotorula mucilaginosa* (syn. *Rhodotorula rubra*), *Rhodotorula minuta* and *Pichia fermentans* (syn. *Candida lambica*) [103] were able to transform ferulic acid into 4-vinylguaiacol.

## 3. Conclusions

Flavors play a key role in the quality perception of food and beverages, whereas fragrances represent an important ingredient in the soap and perfume industry. For many years, most of them were extracted from natural sources or produced via chemical synthesis. The rising consumer requests for natural flavors and fragrances have generated a great interest in the aroma industry to seek new methods to obtain fragrance and flavor compounds naturally. An alternative and attractive route for these compounds is based on microbial biotransformations.

In this review, we summarized several examples where NCYs whole cells used as biocatalysts can be considered a complementary and cheap system for the production of flavors and fragrances. The use of environmentally-friendly conditions (e.g., the use of mild reaction conditions, *i.e.*, the absence of expensive and sometimes dangerous reagents or extreme working temperatures) makes the use of NCYs whole cells as an excellent substitute of some chemical conventional processes. These examples indicate that NCYs can be considered a safe method for the production of flavors and fragrances. Of course, the chemical variety of flavors and fragrances might match the wide (and ever increasing) biological and metabolic diversity of NCYs. So, the number of novel processes still to be explored and developed is most likely to increase noticeably in the coming years. This will probably led to a more productive exploitation of these biotransformation procedures. Furthermore, biotransformation by NCYs may represent in the future a powerful tool to optimize the biological (e.g., bioactivity, olfactory properties, *etc.*) and the physico-chemical properties (e.g., solubility, lipophilicicty, *etc.*) of flavors and fragrances, as recently reported [104].

In addition to conversion of precursor-compounds (biotransformation), another potential source of flavors and fragrances is the bio-synthesis by microorganisms, which represent a highly attractive way to produce known or novel flavors. In this context, yeast biodiversity may be greatly impacted by synthetic biology, and yeast biotechnology has thus gained great interest in the last decades: the production of different valuable products of primary and secondary metabolism could be enabled by joining the potentials of genomics, metabolic engineering, systems and synthetic biology. Engineering of yeasts as natural production hosts could improve the number of valuable flavors and fragrances, and also their productivity, as the increasing availability of yeast genome sequences enables better understanding and relief of rate-limiting steps based on the results of omics data and metabolic modeling. Thus, the enzymes responsible for the biosynthesis of flavor and fragrance molecules or of its precursor may be cloned and characterized, and the biosynthetic pathway reconstructed in genetically engineered yeasts, analogously to what was performed by Schalk *et al.*, which have reconstructed the sclareol biosynthetic pathway in genetically engineered *Escherichia coli* [105].

Currently, some companies like Givaudan, Firmenich, and International Flavors & Fragrances are involved in producing “biotech” perfumes.

Clearwood™, a mixture of sesquiterpenes and alcohols present in patchouli oil, is a biotech natural fragrance ingredient rich in patchoulol, introduced into the market in 2014 by Firmenich, the Swiss purveyor of flavors and fragrances. Patchoulol has been produced through synthetically altered microorganisms by California-based biotech company Amyris in partnership with Firmenich. The companies have developed a novel bioprocess for producing large, quality volumes of patchouli oil from yeast and are currently doing so in Amyris’s facility in Brotas, Brazil [106].

Also in 2014, Givaudan introduced Akigalawood^®^, a fragrance ingredient obtained through an enzymatic process to transform a specific fraction of the natural raw material Patchouli into a new, natural and captive perfume compound [107].
